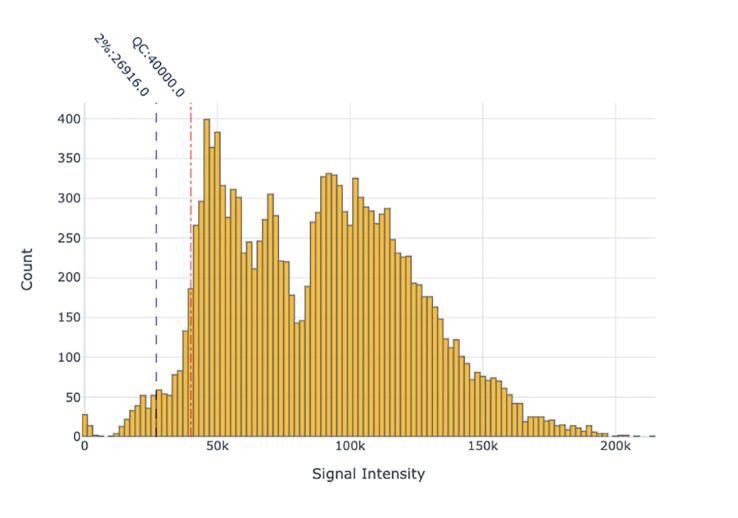# Listening to your mass spectrometer: An open-source toolkit to visualize mass spectrometer data

**DOI:** 10.1016/j.jmsacl.2021.12.003

**Published:** 2021-12-13

**Authors:** Abed Pablo, Andrew N. Hoofnagle, Patrick C. Mathias

**Affiliations:** Department of Laboratory Medicine and Pathology, University of Washington School of Medicine, Seattle, WA, USA; Department of Laboratory Medicine and Pathology, 1959 NE Pacific St, University of Washington, Seattle, WA 98195-7110, USA

**Keywords:** GB, Gigabyte, LC-MS/MS, Liquid chromatography tandem mass spectrometry, LLOQ, Lower limit of quantification, MB, Megabyte, QC, Quality control, RRT, Relative retention time, Python, Dashboard, Database, Visualization, Quality control, Mass spectrometry

## Abstract

**Introduction:**

We have developed a set of tools built with open-source software that includes both a database and a visualization component to collect LC-MS/MS data and monitor quality control parameters.

**Description of tool:**

To display LC-MS/MS data we built a parsing tool using Python and standard libraries to parse the XML files after each clinical run. The tool parses the necessary information to store a database comprised of three distinct tables. Another component to this toolkit is an interactive data visualization tool that uses the data from the database. There are 5 different visualizations that present the data based on interchangeable parameters.

**Evaluation of tool:**

Using histogram visualization, we assessed how quality control parameters that feed our quality control algorithm, SMACK, which assists to improve the efficiency of data review and results, performed against the collective data. Using the newly identified QC parameter values from the toolkit, we compared the output of the SMACK algorithm; the number of QC flags changed in that there was a 1.7% (31/1944 observations) increase in flags and a 7.1% (138/1944 observations) decrease in presumed false positive flags, increasing the overall performance of SMACK which helped staff focus their time on reviewing more concerning QC failures.

**Discussion:**

We have developed a customizable web-based dashboard for instrument performance monitoring for our opiate confirmation LC-MS/MS assay using data collected with each batch. The web-based platform allows users to monitor instrument performance and can encompass other instruments throughout the laboratory. This information can help the laboratory take proactive measures to maintain instruments, ultimately reducing the amount down time needed for maintenance.

## Highlights

• Mass spectrometry produces data which can be used to monitor instrument performance.
• We describe a tool that parses and visualizes mass spectrometry data.
• This toolkit can be applied to increase quality control for a complex LCMS assay.

## Introduction

Liquid chromatography-tandem mass spectrometry (LC-MS/MS) is an established testing method in clinical laboratories providing toxicology services. The technique’s accuracy and throughput provide great advantages for analysis of small molecules. Every injection from an LC-MS/MS system is coupled with a wealth of instrument metadata that can be used to evaluate the acceptability of a given assay. As the size of data sets increase, there becomes a valuable opportunity to analyze the data for clinical and practical purposes on a large scale. In order to make such analyses possible, tools are needed for individual laboratories to use, explore, and mine large scale data sets.

Laboratories have developed and implemented dashboard tools to monitor and track, optimize, and support specialized laboratory workflows that are not always well-supported by native laboratory information system functionality [1], [2], [3], [4], [5]. Outside of the clinical laboratory space, a number of tools focused on proteomics have been developed to assess LC-MS-based data quality, incorporating instrument performance [6], [7], [8]. Slade et. al [9] describe a similar tool used in the laboratory to gather and display instrument data with an interactive dashboard. As the complexity of LC-MS/MS assays increase, performing quality control assessment of data offers significant advantages, including identification of technical variability derived from sample collection, preparation, and/or instrument performance.

The University of Washington Department of Laboratory Medicine and Pathology uses mass spectrometry in several testing contexts, including therapeutic drug monitoring [10], [11], protein biomarker quantification [12], [13], [14], vitamin measurement [15], [16], [17], and monitoring of prescription pain medication. The most complex application of mass spectrometry in our setting is a dilute-and-shoot quantitative/semi-quantitative LC-MS/MS confirmation assay that measures 12 opioids and 11 metabolites [18], performed on Waters Xevo TQ-MS instruments. Pathologists on the Clinical Chemistry service then integrate results from the assay with information from the patient’s chart to provide interpretations for the results. These results and interpretations are used to support decisions to continue or discontinue patient opioid therapy, and, therefore, robust quality control metrics are critical to the assay's clinical utility. To manage the complex workflow and data analysis process, our team previously developed software for automated quality control and data analysis for this opiate LC-MS/MS assay [19]. The software helped to improve the consistency of data analysis and reduce the amount of time technologists spent reviewing the data. The software identified any analyte that failed QC, which was then manually reviewed. There were a number of quality control metrics such as relative retention time of analytes and internal standard peak areas, for each patient specimen that required review. The software, “SMACK”, was developed as a command line application written in Python that ingests an XML file from the Waters Target Lynx application used to review chromatographic data and performs quality control calculations. The SMACK program contains algorithms that compare assay data to limits of detection and limits of quantification gathered during the validation of the assay to improve the efficiency of data review and resulting. The SMACK program assesses RRT, internal standard peak area cutoff, a cutoff peak area of the first calibrator, signal-to-noise cutoff, and ion ratio cutoffs.

The assay protocol has evolved with new compounds and internal standards having been added to the assay over time. Currently, there are 24 compounds and 24 internal standards. In 2020, the laboratory tested hundreds of samples a week, splitting the load between instruments. As the assay has evolved, it is important to ensure the QC parameters used to drive manual review of data are aligned with the expected values based on historical data. For these reasons, along with the nature of a dilute-and-shoot sample preparation method, QC limit assessment and instrument performance monitoring are critical to an efficient assay workflow. A challenge for many laboratories is having the ability to easily access metadata in a unified, central location. Not all MS instrument middleware support the ability to flexibly review relevant historical data. Purpose-built middleware may be costly or may limit data-review to onsite access only. Also, laboratory information systems often store sample QC and result values only, excluding valuable data.

To address our data review and quality needs, we developed a set of tools built with open-source software that includes both a database and a visualization component to collect LC-MS/MS data and monitor quality assurance parameters. These tools assist with automated quality control and data analysis for our complex LC-MS/MS assay for urine opiates and metabolites, and enable our team to continuously monitor and update the SMACK application QC parameters.

## Description

**Supplementary figure 1:**
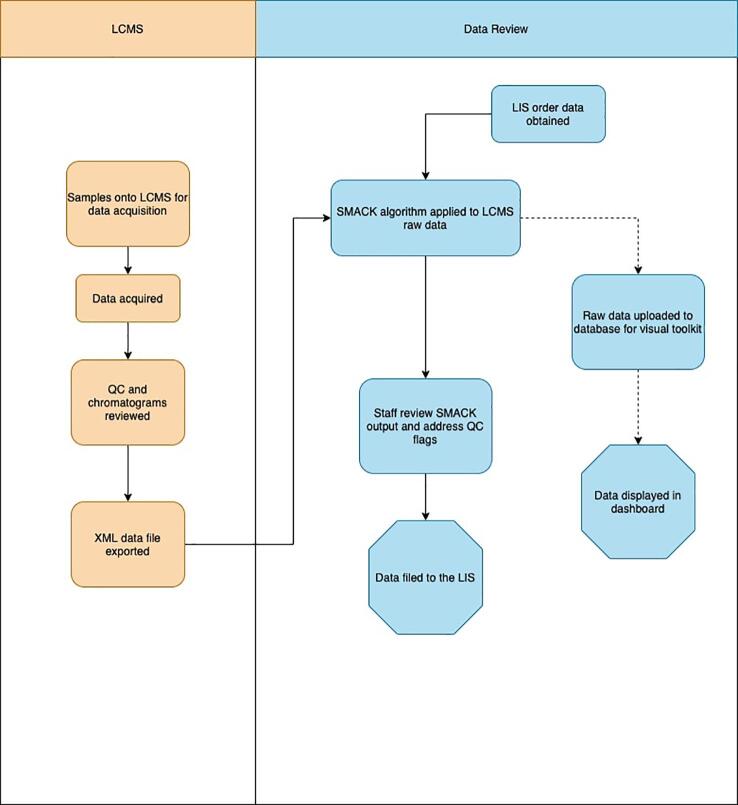


One important component of this quality tool is a centralized and secure database to store all LC-MS/MS raw data using PostgreSQL, an open-source relational database management system. The database stores data in three separate tables (Table 1). A batch level table includes number of samples, instrument identification, and name of the XML files. A calibration level table includes the equation of the calibration curve by compound, the weighting on the curve equation, and r-square value. Finally, a result level table includes the sample type, sample name, vial position, batch file name, and compound information such as peak area, RRT, etc. This design was inspired by the data model used by Indigo BioAutomation in ASCENT, a commercial product used for automated mass spectrometry data review (www.indigobio.com/ascent). The toolkit was developed to be incorporated into the laboratory workflow. Supplementary Fig. 1 provides a visual schematic outlining the flow of data in relation to the user review process.

**Table 1:** Database table schema. Three tables, ‘batch’, ‘calibration’, and ‘results’ hold the metadata for each batch that was parsed from the XML file. Each row in each table is assigned a primary key that is unique and acts as a row identifier .

Table	batch	calibration		results		
Columns	batch primary key	calibration primary key	Weighting	results primary key	Sample Type	Confirming Ion Area
	XML file name	XML file name	Slope	XML file name	Compound	Internal Standard ID
	Timestamp	Timestamp	R-Squared	LC Batch Name	Compound ID	Internal Standard
	Instrument ID	Compound	CC (Continuing Calibration)	Injection Time	Peak Area	IS Peak Area
	Number of Samples	Curve Type	Internal Standard ID	Vial	Concentration	IS RT
		Origin	Internal Standard	Sample	RT	IS SN
				Sample ID	SN	IS Confirming Ion Area

To load the data into the database, a parsing tool was developed as part of the toolkit. The tool was developed using Python with standard and open-source libraries, such as Pandas, NumPy, and SQLAlchemy. The tool's input is an XML file or a folder containing several XML files. The tool automatically reads each file then parses out the relevant information and organizes the data in a tabular format that populates each database table.

Another important component of the toolkit is an interactive data visualization tool that uses the data from the PostgreSQL database. The data visualization tool was built using the Dash open-source library from Plotly. We designed 5 different figures (Table 2) that visualize and summarize the quality control data, filtered by various metadata elements. A mock-live version of the quality control monitoring dashboard supplied with 2 week's worth of mock data can be found at the following URL (https://desolate-citadel-06032.herokuapp.com/).

**Table 2:** Visualization Tool Summary. Each visualization view plots or displays data in a different manner. Each column lists the interchangeable variables to specify separate data analysis views. The summary tables adjust as each variable is changed in the visualization.

Histogram	Plotted Average	Plotted Batch	Plotted Std-A Signal	Absolute RT vs Monthly Average
Instrument	Instrument	Instrument	Instrument	Date
Sample Type	Timeframe	QC Parameter	Batch	Instrument
QC Parameter	QC Parameter	Compound	Compound	Color code table summary
Compound	Sample Type	Sample Type	Month/Number Cumulation	
Date Range	Compound	Batch		
	Statistical Summary Table	Statistical Summary Table		

The historical histograms provide a way to view instrument data collected across a set time period. The average plotted performance provides a closer statistical view of the data split by specific time periods. The plotted batch data provides a view of instrument performance for a given batch. The plotted Std-A signal provides one view to monitor signal performance from batch-to-batch. The absolute RT provides a direct statistical view of the chromatography performance from batch-to-batch for a set time period. Using these figures, the laboratory should be able to catch potential mass spectrometer and/or chromatography issues and act swiftly.

The complete Git repository including source code and documentation is available at https://github.com/pablouw/opiateDashboard.

## Evaluation

The parameters that the SMACK application uses to evaluate assay results to determine result acceptance or failure are RRT, internal standard peak area cutoff, a cutoff peak area of the first calibrator, signal-to-noise cutoff, and ion ratio cutoffs. The values of each cutoff, or window, for each compound were obtained during validation. Using the histogram graph of the toolkit, we plotted the metadata for each compound for each of the two mass spectrometers. We evaluated the historical data using both the quality control materials and calibrators only, and then including the patient data. We also plotted the value of the cutoff of each parameter for comparison. Also, we plotted the calculated values that represented the borders that captured where the bottom 2% of data were excluded.

**Fig. 1:**
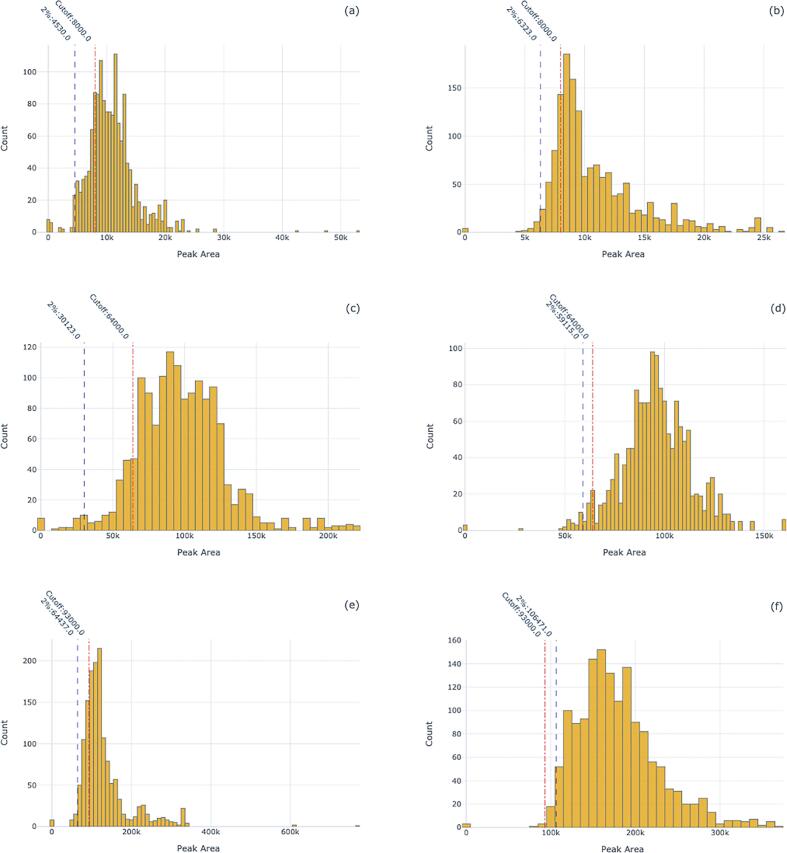
Internal Standard Signal of Calibrators Across Instruments. Figures a, c, e show distributions of instrument 1, and b, d, f show distributions of instrument 2. Figures a, b display the distributions of fentanyl-d_5_. Figures c, d display the distributions of methadone-d_9_. Figures e, f display the distributions of normeperidine-d_4_. Each plot shows the current cutoff value for each internal standard relative to the distribution of the internal standard signal for the calibrators along the value where the bottom 2% of data was excluded. The percent of samples below the QC cutoff are 21%(a) (n = 1,460), 17%(b) (n = 1,489), 10%(c) (n = 1,460), 4%(d) (n = 1,489), 21%(e) (n = 1,460), and 0.5%(f) (n = 1,489).

By using this graph and values we wanted to assess how the provided cutoff values and ranges programmed into the SMACK program performed for each instrument over time. Graphing the control and calibrator data only, we found that for three compounds the internal standard cutoffs that were provided to the SMACK application were too high for a particular instrument. Fig. 1 displays the cutoff value relative to the distribution of the internal standard. We also uncovered variation in the performance of these cutoffs between the different instruments on which these assays were performed. For the remaining internal standard cutoffs, the values were not high enough.

**Fig. 2:**
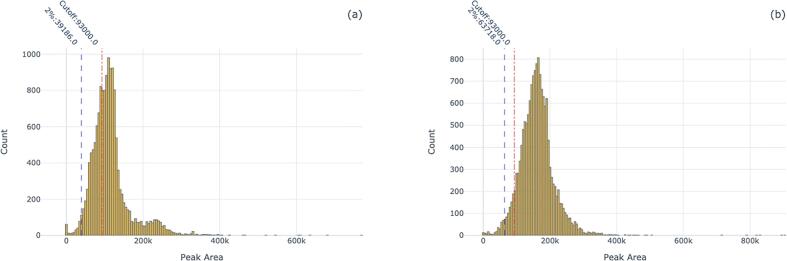
Distribution of Normeperidine-d_4_ internal standard on two separate LC-MS/MS instruments. The proportions of samples below the cutoff was 34.22% for instrument 1 (Fig. 1a, n = 14,589) and 6.79% for instrument 2 (Fig. 1b, n = 15,263).

We expected that including patient data would provide a more realistic view of how the cutoffs performed due to the presence of matrix effects. Plotting patient data demonstrated that our internal standard cutoffs performed fairly well for one instrument, but not the other. Fig. 2 shows the distribution of the internal standard Normeperidine-d4 on both MS instruments.

We also saw bimodal distributions in some instances. Supplemental Fig. 2 displays the distribution of the internal standard EDDP-d_3_ as seen on instrument 1. Two possibilities could explain these instances. One, by using the raw data we consider noise that would be neglected if any concentration value was under our LLOQ by the SMACK software. Second, the quality of the sample may not be neglected. Interfering substances may be present in the sample which affects the assay's precision and accuracy of the analyte.

**Fig. 3:**
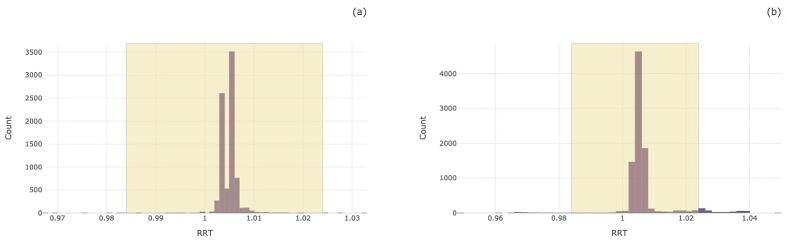
Distribution of Relative Retention Time of 6-Monoacetlymorphine relative to 6-Monoacetlymorphine-d_6_. For both instruments the window ranged from 0.984 to 1.024 where 0.12% of samples fell outside of the window for instrument 1 (a) (n = 8,039) and 4.71% fell outside the window for instrument 2 (b) (n = 8,940).

Our results also show that the RRT range used in the 'SMACK' software was wide and that making it more stringent would improve the rate at which we capture false-positives for the majority of the compounds. When comparing the performance of the LC system, the elution time was mostly in agreement apart from a couple of compounds, varying slightly depending on the instrument. Fig. 3 displays the RRT distribution of 6-Monoacetylmorphine on both LCMS instruments. We would expect the compound and internal standard to co-elute, therefore, we would expect the RRT to be roughly 1.0. However, the quality of the urine specimen cannot be excluded, and interfering substances can be factors.

From this data, we updated the parameters of the SMACK program. We then compared this new version with the previous version by assessing the QC flag output of the SMACK application. We used the same integrated XML files from different days, being sure to vary the technologist and instrument. Comparing the outputs, the number of QC flags changed in that there was a 1.7% (31/1944 observations) increase in flags and a 7.1% (138/1944 observations) decrease in presumed false positive flags. Flags help draw attention to reviewing staff for potentially problematic samples. The difference in the number of flags helped in the overall performance of SMACK which helped staff focus their time on reviewing questionable QC failures, rather than good-quality peaks that were raised as QC flags because they bordered the cutoff values and ranges.

## Discussion

Using tools developed with open-source software, we parsed and collected valuable LC-MS/MS data and built a dashboard that summarized the data in different graphical forms. From these interactive graphs, we assessed the values feeding our quality control algorithm and evaluated the performance of those values before and after making changes to the algorithm. Our assessment revealed that the values were not a great fit, and by updating the values we were able to improve upon the rate of false-positives and QC failures.

We built this tool kit to collect and review data that was otherwise not fully utilized. We chose to use a SQL database for the many customizable options and features it provides. One feature is that one can validate data on import to help keep data consistent. For instance, one can restrict data sources to make sure only numeric-type values are permitted. We had found many files that had missing pieces of data. For our purposes, we opted to accept this data as null. The application did not use these values in the calculations for the visualizations and statistics. Another feature is that the database is not tied to one instrument. This assay is spread across two instruments and can be expanded to include more without the need to rewrite any of the code.

Additionally, a database can protect your data by limiting the access to the data to select personnel and even allows multiple people to view data simultaneously from multiple computers, something that is not an option with Microsoft Excel. Many laboratories use spreadsheets to maintain and work with historical data. Maintaining a spreadsheet to assess data only works for a limited volume of data. If the number of observations exceeds one million, spreadsheets become slow, unresponsive, or may not allow the data set to be collected in a single file. Utilizing a database reduced the storage footprint from 11 GB to 170 MB, a 64-fold decrease. While spreadsheets can be password protected, this is not standard practice. Also, spreadsheets are often limited to one person being able to edit the file. With a database, multiple users can edit simultaneously, and edits can be tracked. Our data implementation restricts user privileges to read only. We wanted to preserve the data as it was and there was no need for users to have the ability to edit historical data. Finally, the database can be stored in multiple locations. We chose to store the database, and the toolkit, on a protected server to allow users to access the toolkit from any computer with internet access without the need to download any software to a computer.

We chose to display data summaries in a graphical format to allow for rapid assessment. The layout for the dashboard was chosen to provide all of the relevant data for a given figure clearly and concisely. Providing data-dense visualizations allows a large amount of information to be displayed within a limited and organized space. The graphs help identify problems that would not be provided by numerical statistical summaries alone. During the design stages, we worked with the laboratory to create and design the figures of the dashboard. Each graph was built with a specific purpose and to help provide the information in a quick and efficient manner. Additionally, we created and designed the figures in stepwise fashion. We began our investigation of the QC parameters after the histogram figure had been created. The figures that were then added were placed for future and ongoing investigations.

We found differences in internal standard peak area between instruments and updated the cutoff values in the algorithm. We have observed different peak areas on different instruments for many years, which we assume is due to differences in ion optics and detector gain settings. For this reason, we added the flexibility of the software application to be able to account for between-instrument variability in internal standard peak areas.

Instrument performance changes over time are expected. The QC parameter values were gathered during validation when the assay was first developed. Additional variables need to be taken into account when considering the baseline data set. For example, the internal standard solution was made in-lab, and slight variations can arise between batches. We arbitrarily accept a 10% bias between batches of internal standard. This provides a sensitive cutoff for worrisome ion suppression in a particular sample during production. When the batch is outside of the 10% threshold, the peak area in the software application is adjusted to avoid significantly altering sensitivity or sensitivity of the cutoff. Moreover, since the assay was first developed, additional compounds have been added and ion enhancement was evaluated. The QC parameter values we evaluate for each clinical sample are relative to each batch and drive the decision of further investigation. Therefore, slight variation is acceptable. For any laboratory investigating the overall instrument performance, the baseline data set should be carefully considered. Finally, SMACK flags analytes that fail QC, and staff review these, along with the overall output, manually. Updating the parameters helps reduce the amount of time staff spends reviewing false positives.

We chose to code the software in Python, a programming language which emphasizes readability. In addition to being a common general purpose programming language, Python is the default language for our departmental informatics team, so it is inherently easier to distribute support needs across our staff. Our organization also frequently uses R for data visualization, and R was a good candidate for this use case; however the staff supportability and our organization’s familiarity with deploying Python-based web applications ultimately nudged us in that direction. We chose the Dash library from Plotly for its ability to create interactive and dynamic graphs, which allows users to more closely inspect the data by selecting ranges and zooming into sections of plots while displaying current QC parameters to compare against historical data. Additionally, the package provides a simple platform to allow users to create custom user interfaces while also simplifying the framework required to build an interactive web-based application. Also, we chose to work with Plotly because of its simplified support to install on different hosts. Security permissions can be handled by Plotly or by the host.

The use of a relational database and Python libraries requires the expertise of experienced programmers and dedicated time to program. While Python is a beginner-friendly programming language, it stills requires time to learn and a specific skill set in order to develop and maintain solutions. However, the practical benefits offered by the dashboard to the laboratory, in terms of medical laboratory scientist time saved, helps increase the return on investment. By collecting and displaying instrument performance information on a routine basis there is also a return on reducing the amount of time required in troubleshooting the instrument. This information can help the laboratory take proactive measures to maintain instruments, ultimately reducing the amount down time needed for instrument maintenance while maintaining high quality performance. The ability to split data by instrument allows the laboratory to assess and compare LC-MS/MS instrument performance. The web-based platform allows users to monitor instrument performance outside of the laboratory and allows for collaboration from multiple parties. The benefits of the dashboard can extend beyond this assay and into other instruments throughout our laboratory and department. In Slade et al [9], the authors describe a tool that collects and stores LCMS batch data in a secure data center which is then accessed on a desktop application built with the R programming language. The tool described here is similar, in that interactive dashboards were constructed for dynamic analysis to explore instrument performance and laboratory workflows. Our tool similarly provides the capability to review historical data along with QC cutoff values. In our setting, this tool and associated visualizations was not intended to support batch-level QC review; rather this data visualization capability helps inform a separate software module we use to aid in the QC process. Applying the tool for this use case (assuring efficiency of our software QC tool) has been uncontroversial. As with other improvement projects in the laboratory, we find that there is a significant amount of change management associated with adopting new data visualization tools to more broadly impact laboratory workflows; thus far, our tool has not yet been transitioned for use in production as a daily batch-level QC tool, although it would function well in that capacity.

In conclusion we have developed a customizable web-based dashboard for instrument and QC software performance monitoring for our opiate confirmation LC-MS/MS assay using the data that is collected with each batch. Dynamic, web-based dashboards are an additional tool laboratories can utilize when commercial options are not readily available or not fit for purpose. Data visualization tools are important for efficiently utilizing data-rich testing methodologies, such as LC-MS/MS, and can help maintain high quality instrument and assay performance.

## Funding

This research did not receive any specific grant from funding agencies in the public, commercial, or not-for-profit sectors.

### CRediT authorship contribution statement

**Abed Pablo:** Conceptualization, Software, Validation, Formal analysis, Writing – original draft. **Andrew N. Hoofnagle:** Writing – review & editing. **Patrick C. Mathias:** Conceptualization, Methodology, Writing – review & editing, Supervision.

## Declaration of Competing Interest

The authors declare that they have no known competing financial interests or personal relationships that could have appeared to influence the work reported in this paper.

## Figures and Tables

**Supplementary figure 2:**