# Supplementary material for: Listening to your mass spectrometer: An open-source toolkit to visualize mass spectrometer data
Source: J Mass Spectrom Adv Clin Lab. 2021 Dec 13;23:44–9. doi: 10.1016/j.jmsacl.2021.12.003 (PMC8739458; doi:10.1016/j.jmsacl.2021.12.003)
Supplement: Supplementary data 1 [file mmc1.docx]

**Supplemental Materials**

| **Package** | **Version** | **Package** | **Version** | **Package** | **Version** |
| --- | --- | --- | --- | --- | --- |
| appnope | 0.1.2 | ipython | 7.24.1 | pickleshare | 0.7.5 |
| argon2-cffi | 20.1.0 | ipython-genutils | 0.2.0 | plotly | 4.14.3 |
| async-generator | 1.1 | ipywidgets | 7.6.3 | prometheus-client | 0.11.0 |
| attrs | 21.2.0 | itsdangerous | 2.0.1 | prompt-toolkit | 3.0.18 |
| backcall | 0.2.0 | jedi | 0.18.0 | ptyprocess | 0.7.0 |
| bleach | 3.3.0 | Jinja2 | 3.0.1 | pycparser | 2.2 |
| Brotli | 1.0.9 | jsonschema | 3.2.0 | Pygments | 2.9.0 |
| cffi | 1.14.5 | jupyter-client | 6.1.12 | pyparsing | 2.4.7 |
| click | 8.0.1 | jupyter-core | 4.7.1 | pyrsistent | 0.17.3 |
| colorlover | 0.3.0 | jupyterlab-pygments | 0.1.2 | python-dateutil | 2.8.1 |
| cufflinks | 0.17.3 | jupyterlab-widgets | 1.0.0 | pytz | 2021.1 |
| dash | 1.20.0 | MarkupSafe | 2.0.1 | pyzmq | 22.1.0 |
| dash-core-components | 1.16.0 | matplotlib-inline | 0.1.2 | retrying | 1.3.3 |
| dash-daq | 0.5.0 | mistune | 0.8.4 | Send2Trash | 1.5.0 |
| dash-html-components | 1.1.3 | nbclient | 0.5.3 | six | 1.16.0 |
| dash-renderer | 1.9.1 | nbconvert | 6.0.7 | terminado | 0.10.1 |
| dash-table | 4.11.3 | nbformat | 5.1.3 | testpath | 0.5.0 |
| decorator | 5.0.9 | nest-asyncio | 1.5.1 | tornado | 6.1 |
| defusedxml | 0.7.1 | notebook | 6.4.0 | traitlets | 5.0.5 |
| entrypoints | 0.3 | numpy | 1.20.3 | wcwidth | 0.2.5 |
| Flask | 2.0.1 | packaging | 20.9 | webencodings | 0.5.1 |
| Flask-Compress | 1.10.0 | pandas | 1.2.4 | Werkzeug | 2.0.1 |
| future | 0.18.2 | pandocfilters | 1.4.3 | widgetsnbextension | 3.5.1 |
| gunicorn | 20.1.0 | parso | 0.8.2 | xlrd | 2.0.1 |
| ipykernel | 5.5.5 | pexpect | 4.8.0 |  |  |

**Supplemental Table 1**. **Required Python Packages.** The list of packages required for the toolkit along with the version of each package.


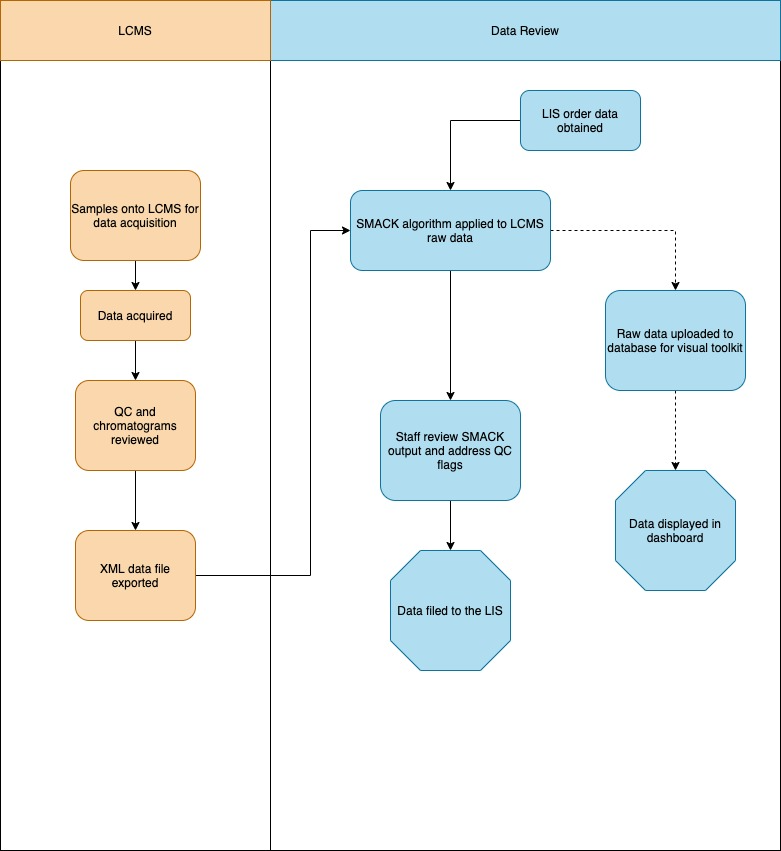


**Supplemental Figure 1. Laboratory Workflow With Toolkit Applied.** When the raw data is uploaded to apply the SMACK algorithm, the toolkit will parse the same data to add to the toolkit database for consumption by the dashboard.


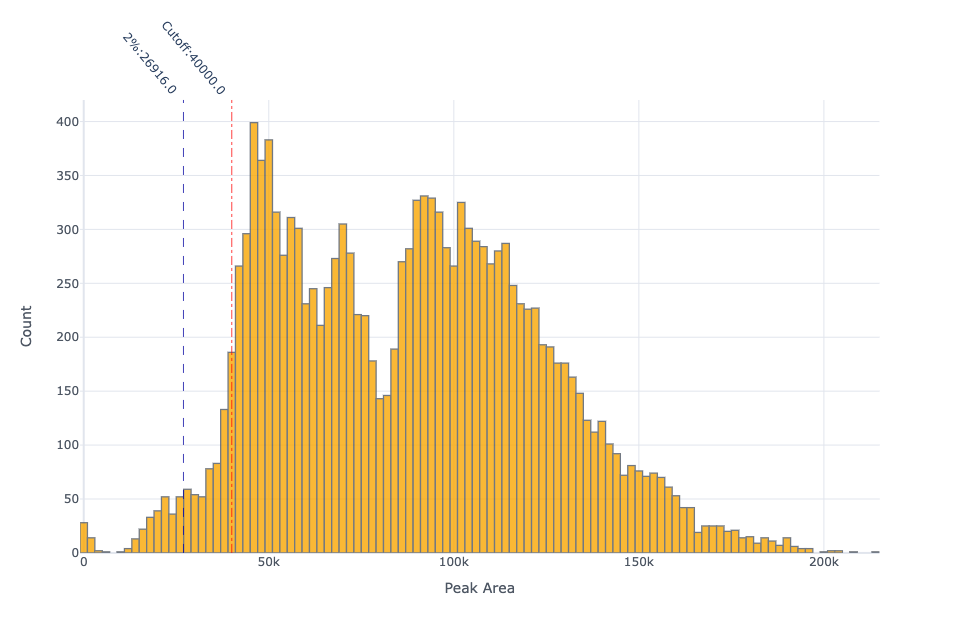


**Supplemental Figure 2. Distribution of EDDP-d3 signal on instrument 1.** The bimodal distribution of the internal standard signal of EDDP-d3 could be affected by matrix effects and sample quality (n = 14,589).
